# Marine heat wave and multiple stressors tip bull kelp forest to sea urchin barrens

**DOI:** 10.1038/s41598-019-51114-y

**Published:** 2019-10-21

**Authors:** L. Rogers-Bennett, C. A. Catton

**Affiliations:** Coastal Marine Science Institute, Karen C. Drayer Wildlife Health Center, University of California, Davis, and California Department of Fish and Wildlife, Bodega Marine Laboratory 2099 Westside Rd., Bodega Bay, CA 94923-0247 USA

**Keywords:** Ecology, Ocean sciences, Community ecology

## Abstract

Extreme climatic events have recently impacted marine ecosystems around the world, including foundation species such as corals and kelps. Here, we describe the rapid climate-driven catastrophic shift in 2014 from a previously robust kelp forest to unproductive large scale urchin barrens in northern California. Bull kelp canopy was reduced by >90% along more than 350 km of coastline. Twenty years of kelp ecosystem surveys reveal the timing and magnitude of events, including mass mortalities of sea stars (2013-), intense ocean warming (2014–2017), and sea urchin barrens (2015-). Multiple stressors led to the unprecedented and long-lasting decline of the kelp forest. Kelp deforestation triggered mass (80%) abalone mortality (2017) resulting in the closure in 2018 of the recreational abalone fishery worth an estimated $44 M and the collapse of the north coast commercial red sea urchin fishery (2015-) worth $3 M. Key questions remain such as the relative roles of ocean warming and sea star disease in the massive purple sea urchin population increase. Science and policy will need to partner to better understand drivers, build climate-resilient fisheries and kelp forest recovery strategies in order to restore essential kelp forest ecosystem services.

## Introduction

Rapid environmental changes are threatening critical marine ecosystems around the world^1^, leading to large-scale catastrophic ecosystem shifts and loss of ecosystem services^2^. Severe declines in key habitat-forming species, or ecosystem engineers, such as corals^3,4^, seagrass^5^ and kelps^6^ will be particularly devastating to biodiversity and productivity. Kelp species are the primary structuring component of highly-productive temperate nearshore rocky reefs^7,8^ growing up to 60 cm per day, but are vulnerable to climate change stressors^9,10^ and may be at risk worldwide^11,12^. Historically, kelp forests have occupied 25% of the world’s coastlines^13^, providing a wide range of ecosystem services, including both habitat structure and food resources^14,15^ as well as modifying light levels and sedimentation^16^, water flow^17^, nutrient dynamics^18^, carbon sequestration^19^ and physical disturbance^20^. Dense kelp beds are biodiversity hot spots, with many kelp-forest obligate species^21^ as well as species utilizing kelp forests as critical nursery habitats^22^, including many economically-important fished species. Kelp forests are resilient to short-term warming events^23^, but multiple severe ecological and climatic stressors could tip kelp ecosystems into an urchin-dominated ecosystem. Sea urchin barrens have multiple feedback loops which could maintain barrens as an alternative stable state^2,24,25^. The dynamics of productive, species-rich, macroalgal-dominated kelp forests are nonlinear and can rapidly transform into unproductive, species-poor urchin-dominated barrens known as a state or phase shift^26–28^.

Starting in 2013, the Northeast Pacific Ocean experienced a record-breaking Marine Heat Wave (MHW) that resulted in well-documented declines of many offshore marine populations and ecosystems, from Baja California to Alaska. Nutrient-poor, warm water conditions associated with the MHW (2013–2015)^29,30^ originated in the Bering Sea, Alaska in 2013 and expanded to the California coast in 2014. Sea surface temperatures 2.5 °C warmer than normal persisted for 226 days, making this MHW the longest duration ever recorded^31^. The MHW led to an unprecedented coast-wide harmful algal bloom which increased concentrations of the neurotoxin domoic acid, resulting in marine mammal strandings and prolonged fishery closures^32^. Further, unusual mass mortality and starvation events were observed in offshore birds and mammals (e.g. Tufted puffin^33^). Overlapping with the MHW, the “Godzilla” El Niño (2015–2016) shifted geographic distributions of warm-water species poleward^34,35^, with unknown impacts to long-term ecosystem community structure and productivity.

**Figure 1:**
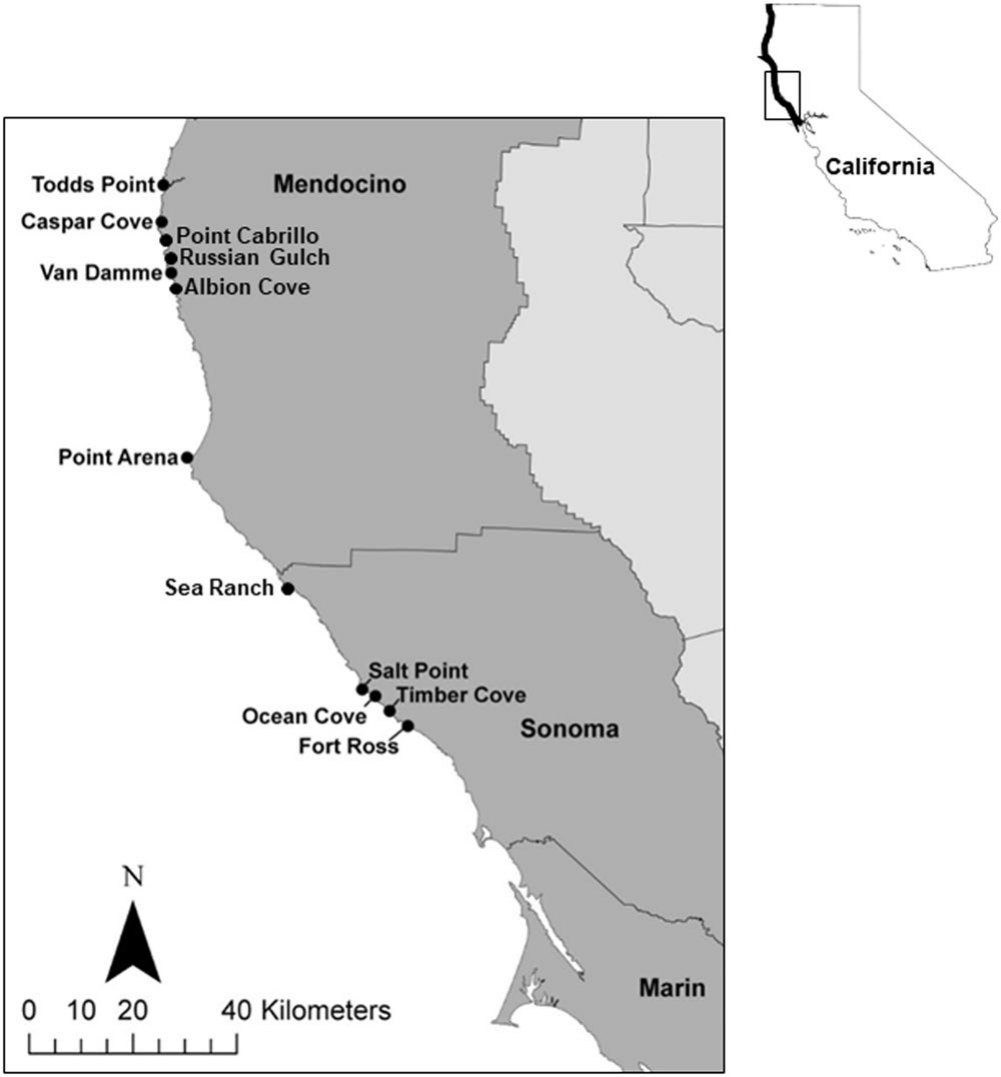
Map of study region in northern California. Extent of aerial survey of kelp canopy represented by the thick black coastline (*inset map*). Subtidal survey sites in Sonoma and Mendocino counties (*main map*). Maps were made using ArcGIS Version 10.6 software by Esri (http://desktop.arcgis.com).

Temperate kelp forests in northern California (Fig. 1) were particularly vulnerable to the MHW and other concurrent ecological stressors. This region, which was historically very productive, supported robust fisheries including the recreational red abalone, *Haliotis rufescens*, fishery (valued at $44 M yr^−1 ^^36^) as well as the commercial red sea urchin, *Mesocentrotus franciscanus*, fishery (valued at $3 M yr^−1^). The bull kelp forests in this region (>350 km) were the first along the west coast of North America to show severe impacts to kelp productivity. The long-term kelp forest monitoring program was critical for tracking and understanding the biological responses to these multiple climate-related stressors and resulting degradation of fisheries and other ecosystem services^37^. Similar impacts seem to be developing in kelp forests from Baja California to Alaska (*personal communications*), so that the dynamics described from this northern California case study will be critical for tracking and understanding the biological responses to these multiple climate-related stressors and resulting degradation of fisheries and other ecosystem services^37^.

Here, we document the catastrophic declines in northern California kelp forests during the MHW, and the subsequent rapid shift of historically persistent kelp ecosystems to wide-spread urchin barrens. We describe the timing and magnitude of events affecting this critical nearshore region based on long-term monitoring data of kelp canopy area (1999–2016), subtidal temperature (2006–2018), and extensive scuba-based ecosystem surveys (1999–2018). We discuss the vulnerability of ecosystem services affecting economic outcomes for the region (e.g. fisheries collapse, loss of tourism), and explore opportunities to enhance resilience^38^ against climate changes which are predicted to increase in the future.

## Results

**Figure 2:**
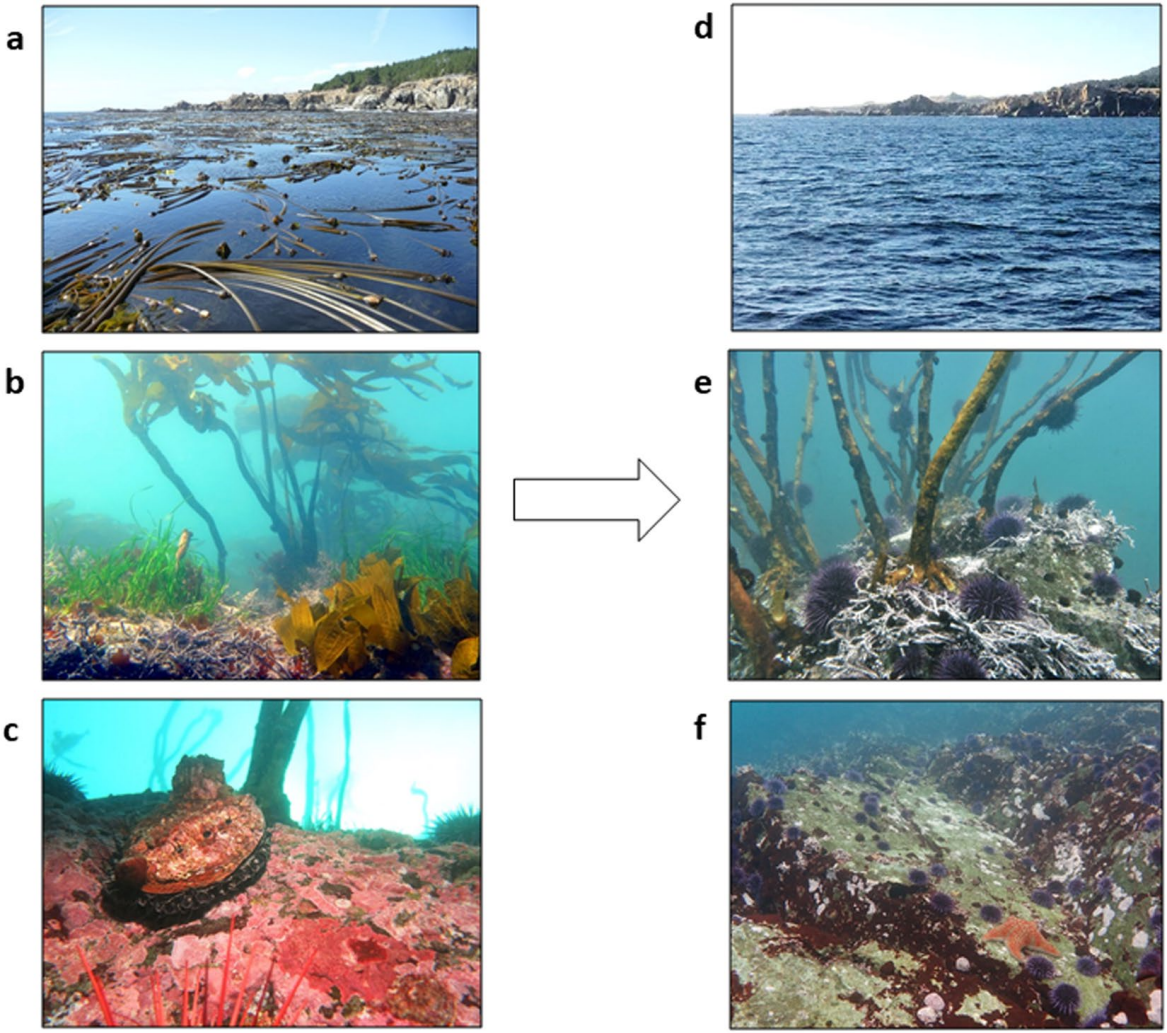
Ecosystem shifts observed for kelp forest canopy *(top)*, subcanopy *(middle*), and benthose (*bottom*), pre-impact (**a–c**) and post-impact (**d–f**). Photo credit: CDFW (K. Joe (**a,c,e**); L. Rogers-Bennett (**b**); C. Catton (**d,f**)).

The region north of San Francisco to the Oregon border (Fig. 1) historically supported extensive, nearly pristine, productive, and persistent bull kelp, *Nereocystis luetkeana*, forests^39^. Human population densities and development are low in the region, so no abrupt anthropogenic impacts to ocean conditions and ecosystem health were anticipated. A series of perturbations^40^ including a loss of sea star predators of urchins^41^, prolonged warm-water conditions, and a population explosion of purple sea urchins occurred prior to and concurrently with an abrupt shift from bull kelp forest to persistent urchin barrens (Fig. 2).

### Bull kelp

**Figure 3:**
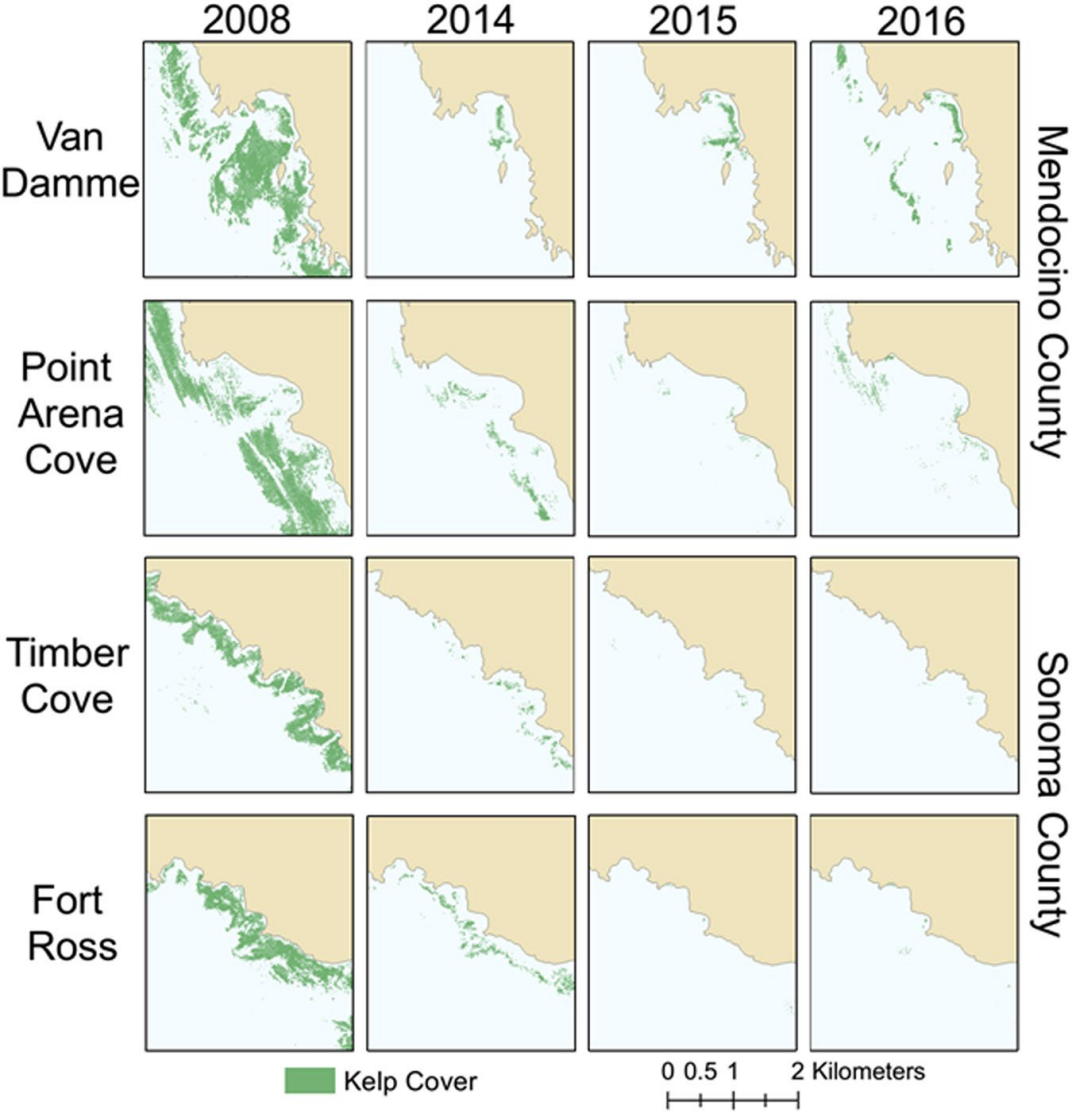
Surface kelp canopy area pre- and post-impact from sites in Sonoma and Mendocino counties, northern California from aerial surveys (2008, 2014–2016). Maps were made using ArcGIS Version 10.6 software by Esri (http://desktop.arcgis.com).

Bull kelp canopy area declined dramatically in 2014 (Fig. 3) throughout the historically-persistent region of bull kelp forest (>350 km of coastline) in northern California. Maximum historic extent of kelp canopy (available data: 1999, 2002, 2003, 2004 and 2008) in the region exceeded 50 km^2^, with a range of 2.4 to 14.9 km^2^ observed in any given year. Nearly 95% of the historic kelp canopy area was observed in Sonoma and Mendocino counties, a 250 km region of coastline dominated by contiguous rocky reef habitat. Bull kelp forests continued to be productive in 2009–2013, growing extensive thick beds throughout Sonoma and Mendocino counties (Fig. 2a; *personal observation*). In 2014–2016, the kelp canopy area declined to <2 km^2^, with no appreciable recovery observed in the core region of the kelp forest in 2017–2019 (*personal observation*).

### Water temperature

**Figure 4:**
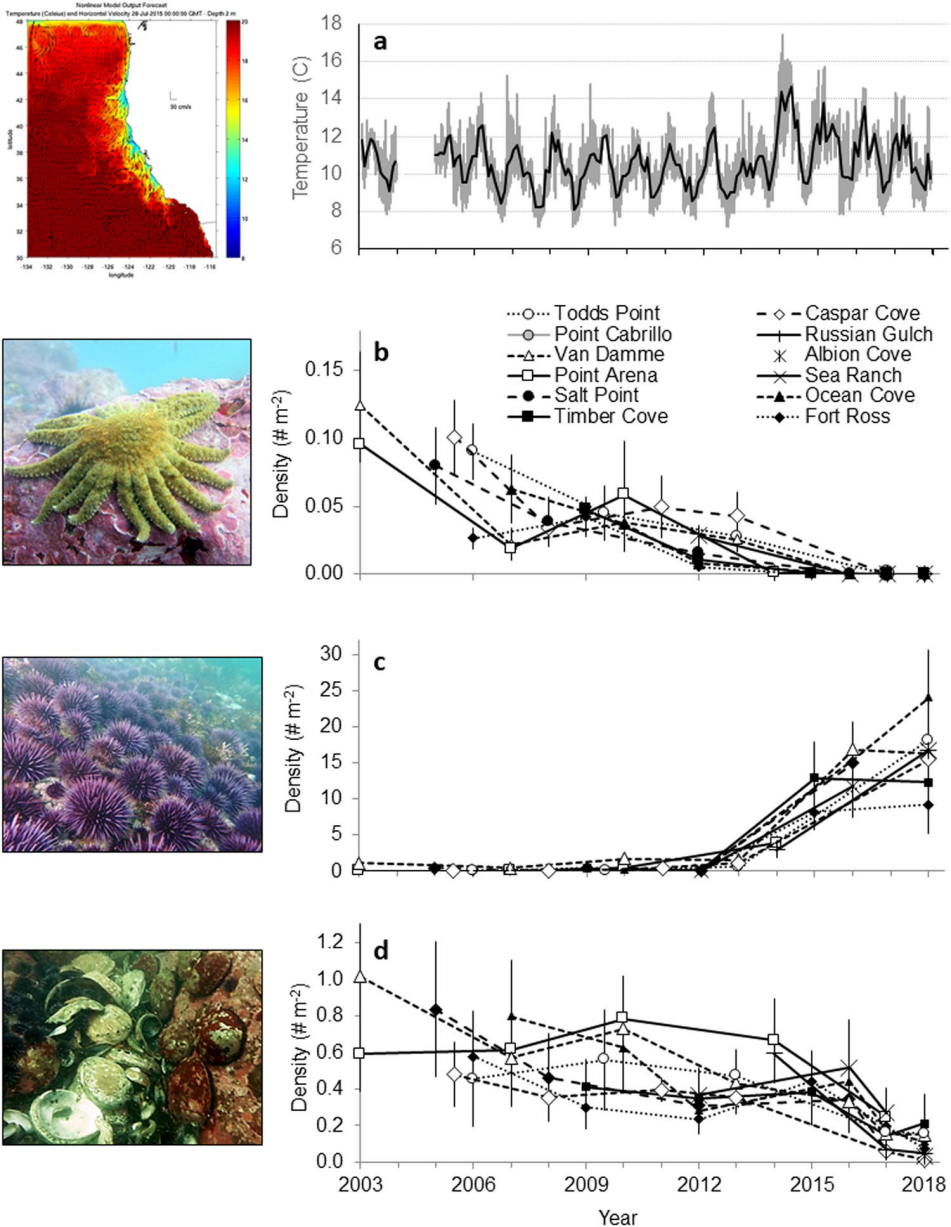
Time series of ecosystem stressors and species abundances (2003–2018). (**a**) Benthic (10 m depth) temperature in Mendocino County; (**b–d**):Average population densities observed across four equal depth strata (0–20 m depth) of Sunflower Stars (**b**), Purple Urchin (**c**), and Red Abalone (**d**). Error bars are s.e.m. across the four depth strata. Image credit: UCSC Ocean Sciences (**a**); CDFW (A. Maguire (**b**), K. Sowul (**c**), K. Joe (**d**)).

The bull kelp decline in 2014 coincided with the onset of the persistent warm water conditions associated with the MHW^30^ in northern California (Fig. 4a Temperature Time Series). Nutrient-poor conditions associated with warmer ocean temperatures (>12 °C)^42,43^ typically appear in fall (September/October), after the primary growing season for bull kelp (June - August). In the summer of 2014 through winter of 2015, daily maximum subtidal nearshore temperatures exceeded 12 °C the majority of days, starting in August (74%) until February 2015 (93%), reaching a record breaking peak temperature of 17.4 °C on September 24, 2014. Cooler temperatures prevailed during the spring upwelling season of 2015, until temperatures exceeding 12 °C again when warm days dominated cool days from July 2015 (65%) to March 2016 (77%). Warmer conditions developed early again in August 2017 and 2018, but were more variable, and on average cooler, than the 2014–2016 time period.

### Sea stars

Prior to the MHW impacts to the kelp forest in northern California, a mass mortality event of twenty seastar species, Sea Star Wasting Syndrome (SSWS)^41,44,45^ decimated local seastar populations from San Mateo to Mendocino counties, beginning in the summer of 2013. Particularly impacted were populations of the Sunflower star, *Pycnopodia helianthoides*, an important urchin predator in kelp forest ecosystems. Prior to 2013, Sunflower stars were commonly observed on transect surveys (average population densities 0.01–0.12 stars m^2^) (Fig. 4b). Within one year of detecting SSWS in the populations, Sunflower stars were functionally extinct (only 1 observed in 2014 and 2015). No Sunflower stars have been observed at any sites 2016–2019, strongly suggesting that this species is now locally extinct.

### Sea urchin

Purple sea urchin, *Strongylocentrotus purpuratus*, were historically very low density in the subtidal (0.0–1.7 urchins m^−2^) prior to 2014, primarily distributed in small dense patches in the shallows. Populations of purple urchins began to moderately increase in the fall of 2014, dramatically increasing 60 fold in 2015 (range: 8.2–12.9 urchins m^−2^) (Fig. 4c). Starting in 2015, the purple sea urchins shifted to a more aggressive feeding behavior associated with food limited urchin barren conditions, grazing down stipes of subcanopy kelps and fleshy algae (Fig. 2e), then grazing through the calcified crustose coralline algal cover (Fig. 2f). Since 2015, purple urchin densities have continued to increase at most of the sites (2018 range: 9.2–24.1 urchins m^−2^).

### Abalone

Red abalone populations were historically abundant (range: 0.24–1.01 abalone m^−2^) and productive prior to the severe ecosystem shifts in 2014 supporting an economically and culturally important fishery. While food-limited conditions progressively worsened after 2014, red abalone populations started to succumb to prolonged starvation, and a mass mortality event initiated in 2017 (Fig. 4d). Piles of shells were observed in the subtidal and severely weakened and shrunken abalone were common. Strong winter storms washed abalone ashore in large numbers, adding to the mass mortality. Population densities decreased at monitoring sites by 48–82% between 2016 and 2017, with additional 43–96% declines observed between 2017 and 2018 (2018 range: 0.01–0.21 abalone m^−2^).

## Discussion

A combination of large-scale environmental and ecological stressors led to dramatically reduced bull kelp canopy in northern California, starting in 2014. Climate-driven impacts of warm-water, including thermal stress and nutrient limitation, associated with the MHW suppressed bull kelp growth (and spore production) during the summer of 2014. These climate-driven impacts persisted for multiple years, and were exacerbated by a strong ecological impact of moderate sea urchin herbivory starting in 2014 and becoming intense in 2015-present. From field observations during subtidal monitoring work, we know that kelp was abundant prior to the impacts in 2014. The continued low bull kelp abundance after 2014 is likely due to the combination of unfavorable environmental conditions (warm water and low nutrients), intensive urchin grazing pressure, and limited spore availability due to multiple years of low production of this annual species.

Starting in 2014, sea urchin populations began to increase to higher densities than previously observed in the region. Populations increased at many sites to more than 30 times historic numbers by 2015, and have continued to increase. Despite widespread starvation conditions, spawning adults of purple urchins have been observed even at sites devoid of macroalgae, and young of the year (<20 mm) are abundant throughout the region. It is unknown if there was a primary driver of the urchin population increase, or if both top-down (sea star predation) and bottom-up recruitment of purple sea urchin processes were responsible. Similarly, the driver(s) of SSWS which led to the local extinction of the Sunflower star is unknown. The first observations of SSWS in the region were recorded during cold-water conditions in the summer of 2013, suggesting that this mass mortality was not initially driven by changes in ocean climate, however warm-water conditions may have later exacerbated the mortalities^44^.

The large-scale ecosystem stressors leading to urchin barrens in northern California illustrates the vulnerability of our ecosystems and communities to climate-driven collapses. Many kelp forest ecosystem services have collapsed on a large scale throughout the region, with particularly severe economic impacts due to collapsed fisheries, kelp harvest, tourism opportunities, and loss of cultural resources. The northern California recreational red abalone fishery was the largest in the world, with 35,000 fishers landing 245,000 abalone (292 mt) yr^−1 ^^36^, however the California and Oregon fisheries were closed in 2018 due to abalone mass mortalities. Widespread abalone starvation and mortality was observed in the wild (Fig. 4d). From previous laboratory experiments, we showed that starvation conditions alone will impact red abalone health and reproduction, which will be exacerbated with warm water^46^. Similarly, the commercial red sea urchin fishery has collapsed due to starvation conditions leading to poor gonad production and unmarketable sea urchins. Small remnant kelp patches (<5%) observed since 2014 are not as capable of promoting kelp recruitment as intact kelp forests^47^. Further, this ecosystem shift to urchin barrens may persist as sea urchins can thrive in low food conditions on dissolved organics as both larvae^48^ and adults^49^ suggesting urchins barrens could be an alternative stable state.

Even if kelps recover from these multiple stressors, it may take decades before the complex biological communities, associates, and the ecosystem services provided by macroalgal forests (Table 1) rebound as has been observed in other parts of the world^50–52^. While the red sea urchin fishery may take only a few months to rebound after kelp recovery, red abalone populations have declined so low that population recovery will likely take decades after kelp populations recover. A host of economically important non-consumptive recreational opportunities, including scuba diving, kayaking, and nature photography, may also impact tourism as the broader nearshore kelp associated community slowly recovers (Table 1).

**Table 1:** Ecosystem services provided by kelp forests to nearshore subtidal marine communities.

Ecosystem Services	Goods and Services	References
Biodiversity	Enhanced Resilience	^11^
	Feeding Habitat	^61^
	Community Structure	^62^
	Enhanced Microorganisms	^63^
Fisheries and Aquaculture	Finfish, Shellfish, Algae	^61, 64^
Recreational Non-Consumptive Activities	Scuba Diving, Kayaking, Photography	^65^
	Economic Source	^66^
Provisioning	Kelp	^67^
	Drift Kelp Subsidies	^68, 69^
	Dissolved Organics	^70^
Coastal Protection	Storm Buffer	^17^
Carbon Sequestration	Oxygen Production	^19^
	Water Quality	^71^
	Fossil Fuel Source	^72^

The documented severe loss of kelp in northern California, starting in 2014, is remarkable because of the scale (>300 km), magnitude (>90%), and speed (within one year) of the impact in an area of historically persistent kelp forests. The severity of on-going ecological and economic consequences underscores the need to investigate the climate impacts and interactions of multiple stressors influencing the vulnerability of ecosystems, even in regions that are relatively pristine (minimal anthropogenic impacts). Identifying the relative impact of individual stressors on a natural system is frequently not possible with observational data alone, particularly when multiple stressors co-occurred or occurred in a rapid sequence. Here, we draw on the long time series of monitoring work and experience with the system, ecological knowledge and theory for kelp forest ecosystems to elucidate the timing of the strongest known stressors in the system.

Given the loss of ecosystem services associated with the shift to an unproductive alternative state, it is important to understand the perturbations that disrupted the marine ecosystem^53^ and its ability to rebound from perturbations (resilience to phase shifts)^4,54^. Identifying the relative importance of factors influencing climate vulnerability is the focus of ongoing research and will be critical for informing recovery potential. A plan for bull kelp recovery in northern California, developed in 2018–2019 with broad scientist and stakeholder input, identifies actionable recovery strategies aimed at enhancing ecological understanding of the drivers that will inform climate ready restoration actions and build resilience for the future^55^.

Science-based management action plans must be initiated to bolster resilience in vulnerable and impacted ecosystems^51^ as all indications are the urchin barrens will persist. In the future, MHW are predicted to continue^56^ increasing in frequency and intensity globally^31^ with the NE Pacific a regional hot-spot^34^. This threat provides strong motivation for developing climate-ready action plans to increase ecosystem resilience to major climate stressors and identify recovery bright spots^57,58^. Such plans should focus on tracking resilience such as within favorable microclimates^59^, enhancing recovery of ecosystem engineers and keystone species, as well as identifying opportunities for economic incentives to support climate resiliency. For kelp forests in California, solutions may include developing economic opportunities to reduce urchin grazing pressure by supporting emerging purple sea urchin restorative fisheries and shifting away from fisheries being the sole support for ecosystem monitoring. Climate-ready resource management will require garnering support and building broad partnerships between science, industry and nonprofits, to develop new monitoring and restoration approaches that enhance resilience of foundational species and their ecosystem services into the future.

## Methods

### Northern california region

We present monitoring data from the nearshore kelp forest ecosystem at sites in rocky subtidal habitats in northern California (San Francisco to the Oregon border), with particular focus on Sonoma and Mendocino counties, from 2003–2018 (Fig. 1). Kelp communities in this region are on rocky reefs dominated by bull kelp, *Nereocystis luetkeana* (Fig. 2a). The understory is comprised of short fleshy red and crustose coralline algae as well as subcanopy kelps, such as *Pterygophora* and *Laminaria* (Fig. 2b). These subtidal rocky reefs in northern California support a diverse assemblage of macroalgae and marine invertebrates.

### Kelp canopy cover

Total kelp surface canopy area was assessed in 2008, 2014–2016 by aerial surveys from San Francisco to the Oregon border. Kelp canopy was quantified using low-flying aircraft to photographically survey the nearshore coastline. Cameras were mounted on the aircraft to capture the images. Image frames were auto-georeferenced using customized software, and manually shifted as needed. ERDAS IMAGINE software was used to mosaic the frames and run them through classification in ERDAS IMAGINE. Maximum extent of the kelp forest was determined by overlaying shapefiles from all available survey years which include 1999, 2002–2005, and 2008. Using ArcGIS Version 10.6 software by Esri, the total area representing kelp on the composite shape file was quantified in km^2^. This procedure shows the potential for kelp canopy cover throughout the area as compared with the extent of canopy cover in a given year. No comparable large-scale kelp aerial survey data exist for this region from 2009–2013.

### Subtidal temperature

Underwater temperature loggers were placed by scuba divers at Van Damme State Park at 10 m depth inside the kelp forest to monitor subsurface sea water temperatures from August 2003–August 2018. Tidbit temperature loggers made by Onset HOBO recorded temperature once per hour and were retrieved once a year by divers in August. Note: There is a gap in August 2004 to October 2005 due to failure of the logger. These data are used to detect the magnitude of the temperature and the frequency and duration of exceedance above 12 °C, an important metric for bull kelp growth as NO_3_ concentrations are low at this temperature and warmer^42^.

### Subtidal scuba surveys

The nearshore kelp forest ecosystem monitoring program^60^ conducts scuba surveys at sites in rocky subtidal habitats along Sonoma and Mendocino counties in northern California. These surveys of the nearshore rocky reefs were initiated in 1999 and allowed for photographic documentation of communities before and after multiple stressors impacted the region. Subtidal surveys were conducted by the California Department of Fish and Wildlife (CDFW) by motor boat at twelve sites along the Sonoma and Mendocino county coasts. The sites ranged in coastal length from 2.4 to 3.2 km. The sites in Sonoma County from south to north include: Fort Ross, Timber Cove, Ocean Cove, Salt Point, and Sea Ranch. In Mendocino County the sites from south to north include: Point Arena, Albion, Van Damme, Russian Gulch, Point Cabrillo (State Marine Reserve), Caspar Cove and Todd’s Point (Fig. 1). The surveys are conducted along band transects 30 × 2 m, located randomly within four depth strata (random stratified) from 1 to 20 m depths. The density estimation for each species is determined by averaging the densities within each of the four depth strata and then calculating the average of the four densities from each depth. The error bars represent standard error of the mean densities across four depth strata. The sites are surveyed to enumerate abalone, sea urchins, sea stars, macro-invertebrate densities as well as percent cover of algae and substrate type. All size classes observed are recorded. At each site 15–55 transects were surveyed. Transects were located in areas with >50% rocky reef.

### Permissions for protected areas

Underwater surveys were conducted inside two marine protected areas. Van Damme State Park (State Marine Conservation Area) and Point Cabrillo (State Marine Reserve) with the permission of the California Department of Fish and Wildlife who is the managing authority for Marine Protected Areas in California.

## Data Availability

The data that support the findings of this study are available from the corresponding author upon request.
